# New Frontiers in 3D Printing Using Biocompatible Polymers

**DOI:** 10.3390/ijms26168016

**Published:** 2025-08-19

**Authors:** Nagireddy Poluri, Jacob Carter, John Grasso, Walter Miller, Matthew Leinbach, Frederick Durant, Riley Benbrook, Assa John, Allan Wang, Xiao Hu

**Affiliations:** 1 Department of Physics and Astronomy, Rowan University, Glassboro, NJ 08028, USA; poluri34@students.rowan.edu (N.P.);; 2 Department of Chemistry and Biochemistry, Rowan University, Glassboro, NJ 08028, USA; 3 Department of Biomedical Engineering, Rowan University, Glassboro, NJ 08028, USA; 4 Department of Biological and Biomedical Sciences, Rowan University, Glassboro, NJ 08028, USA

**Keywords:** 3D printing, biopolymer, bioprinting, crosslinking, nanocomposites, drug delivery, biosensor, tissue regeneration

## Abstract

Biocompatible polymers have emerged as essential materials in medical 3D printing, enabling the fabrication of scaffolds, tissue constructs, drug delivery systems, and biosensors for applications in and on the human body. This review aims to provide a comprehensive overview of the current state of 3D-printable biocompatible polymers and their composites, with an emphasis on their processing methods, properties, and biomedical uses. The scope of this work includes both natural and synthetic biocompatible polymers, polymer–nanocomposite systems, and bioinks that do not require photo initiators. The relevant literature was critically examined to classify materials by type, evaluate their compatibility with major 3D printing techniques such as stereolithography, selective laser sintering, and fused deposition modeling, and assess their performance in various medical applications. Key findings highlight that reinforced polymer composites, tailored surface chemistries, and hybrid printing strategies significantly expand the range of functional, customizable, and affordable biomedical devices. This review concludes by discussing present-day applications and emerging trends, underscoring that 3D-printable biocompatible polymers are rapidly transitioning from research to clinical practice, offering transformative potential for patient-specific healthcare solutions.

## 1. Introduction

Over the past decade, there has been an upwelling of technological advances across various scientific disciplines. Of these storied leaps, few have the potential to impact daily life more than three-dimensional (3D) printing [1]. From its ideological inception in the 1940s to the first implementation in the 1980s, and especially with the advances made during the past decade, this technology has become ubiquitous in laboratories, engineering firms, and even tech-savvy households [1,2]. Three-dimensional printing has a broad range of applications, including but not limited to bioprinting, food printing, metal printing, and ceramic printing [1,2,3,4,5,6,7,8,9,10,11,12,13,14,15,16]. The various methods differ based on the ink or filament used, as well as the physical capabilities of the printer. These materials offer different intrinsic properties to solve a variety of problems, such as varying mechanical strengths or flexibilities, providing the necessary support or malleability where appropriate. The benefits of this customizability are many, allowing for unique solutions tailored to specific problems, rather than relying on the closest commercially available option. These printers allow for fast and precise manufacturing with highly customizable properties [1].

One fast-growing challenge in the medical industry is the increasing demand for organ transplants. The advocacy for organ donation has not kept pace with the growing need. As of 2025, there are over 105,000 candidates on the waitlist to receive an organ transplant, with over 1000 of them being children between the ages of six and ten. In the United States, a child aged six to ten has a median wait time of 137 days for a kidney transplant [1]. There are likely many more who do not qualify or live long enough to become viable candidates. Organs, especially kidneys, are a precious commodity, and there are simply not enough to meet demand [2,3]. Projections show that transplant wait times are only expected to increase [4,5,6], and approximately 10% of patients on a transplant list will die before ever receiving a donor organ [7]. The ever-growing need for organs has been met with a solution that once seemed like science fiction. Three-dimensional printing in the biomedical field gained immense attention when the concept of printing a functional human organ using a recipient’s own cells was introduced. This innovation could dramatically increase the number of available organs and reduce the need for immunosuppressants, since the tissue would be composed of the recipient’s own biomarkers and thus have a much lower risk of rejection. The first 3D-printed organ, a functioning bladder covered in the patient’s own living tissue, was implanted in 1999 [8]. Since that proof of concept, the organ printing field has rapidly expanded, demonstrating the realistic potential for printing and implanting more types of organs.

One of the most significant innovations in medical 3D printing has come through the use of biopolymer-based composite materials. A biopolymer is any polymer synthesized within an organism or created from biological materials outside of a living system. Several biopolymers have seen practical use in the medical field. Cellulose, found in plant cell walls, is one of the most easily accessible biopolymers currently in use. Printing organs and other tissue-related structures, such as heart valves and vascular implants, is not the only medical application of 3D printing. Three-dimensional bioprinting is also being used to fabricate non-living, biocompatible biomaterials such as intraocular lenses, catheters, prosthetics, and other implants in high demand [9,10,11,12,13]. The use of biopolymers and their composites is critical when working with living tissue, as these materials must be biocompatible. Rejection, chemical leaching, and undesirable material properties can compromise an implant’s function. Ensuring that the material does not interfere with tissue development is a fundamental principle in biomaterials science [13].

The technique of 3D printing has also gained popularity in the area of drug delivery systems [14]. It offers considerable advantages due to the ability to tailor compound composition, shape, dosage, and delivery rate. These parameters can be adjusted to maximize therapeutic efficacy [15,16]. As research has advanced, targeted-release methods have been developed and implemented [17]. About 70% of patients are not compatible with standard drug formulations, which underscores the need for individualized treatment pathways. Print porosity can be customized based on age, weight, gender, and other biological factors. Pediatric and geriatric patients particularly benefit from such tailored drug release mapping [18]. This is especially useful for potent drugs with narrow therapeutic windows [19]. In some applications, drug powders are used as ink, allowing pills to be printed directly. This enables more economical and time-efficient drug production [20]. Biomaterials can also be printed layer by layer to form biodegradable scaffolds capable of housing larger molecules for therapeutic delivery [21,22]. These scaffolds play a critical role in drug design and delivery, offering a controlled environment in which drug molecules can develop. They provide structural support and promote molecular growth under precise conditions, facilitating the cultivation of more targeted and effective compounds.

This review outlines the capabilities of 3D printers, the material science behind them, and the opportunities and limitations they present. Despite these exciting advancements, several critical challenges remain to be addressed to fully realize the potential of 3D bioprinting with biopolymers. One major limitation is the development of bio-inks that simultaneously offer printability, mechanical strength, and biocompatibility, which is essential for producing viable and functional tissue constructs [23,24]. Additionally, achieving a high resolution and structural fidelity while maintaining cell viability during the printing process remains a significant technical hurdle. Scalability and reproducibility for clinical and industrial applications continue to be constrained by complex fabrication protocols and a lack of standardized materials [25]. This review aims to address these frontier challenges by synthesizing current material developments, fabrication techniques, and applications. A thorough understanding of biomaterial fabrication history and familiarity with earlier printing techniques, and their technical constraints, is essential for advancing future innovation in the field [26].

## 2. Biocompatible Polymers for 3D Printing

As 3D printing technology becomes more advanced, it enables us to undertake projects that were once impossible. Three-dimensional printing can produce objects ranging from something as small as a keychain to as large as a house. Jewelry, clothing, and robots are all examples of concepts that have been reimagined and brought to life through 3D printing, demonstrating that creativity is the only limit. Arguably, the most impressive achievements of 3D printing have been in the medical field, where organs, tissues, heart valves, and even tracheal substitutes have been successfully printed. Various 3D printing methods make these breakthroughs possible; however, the true foundation of this progress lies in the biomaterials required to bring them to life. All 3D printers require a filament—whether it comes in the form of a spool, powder, or bio-ink. Different biomaterials are selected for specific applications based on their unique properties, and some may require additives to enhance characteristics such as mechanical strength, thermal stability, or biocompatibility.

**Figure 1:**
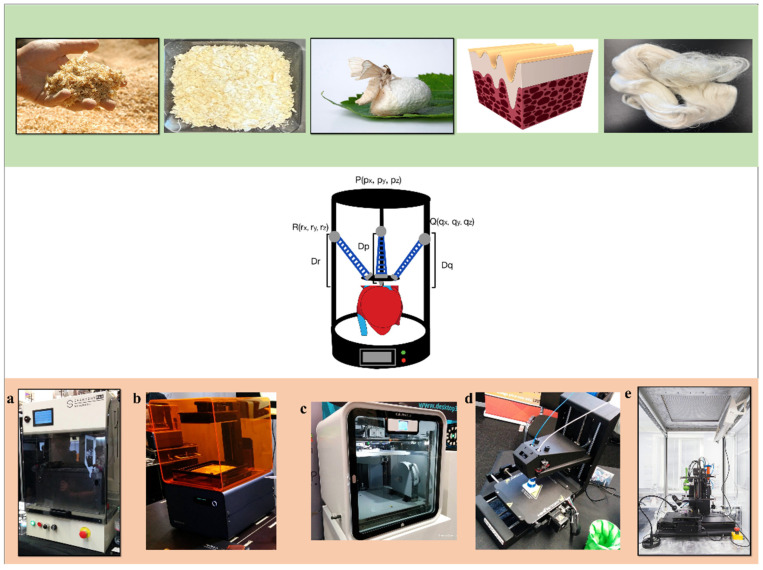
(Top row) 3D-printable biopolymers in raw form, from left to right: cellulose from wood, chitosan from shrimp, silk from silkworms, collagen from skin, and keratin from wool. (Center row) Stylized representation of potential future applications of a 3D printer. (Bottom row) Common 3D printing technologies, including (**a**) selective laser sintering, (**b**) digital light photopolymerization, (**c**) fused deposition modeling, (**d**) fused filament deposition modeling, and (**e**) inkjet printing. (skin, wood, and silkworm photo credits: pixabay.com (2025); “3D Printshow 2014 London-Sharebot SnowWhite SLS 3D printer v01” by Creative Tools is licensed under CC BY 2.0; “3D Printshow 2014 London-Formlabs Form 1 SLA 3D printer” by Creative Tools is licensed under CC BY 2.0; “3D Printshow 2014 London-CubePro v01” by Creative Tools is licensed under CC BY 2.0; “3D Printshow 2014 London-MiniFactory 3D printer v01” by Creative Tools is licensed under CC BY 2.0; “Printer 3D Bioprinting Solutions.jpg” by Aндpeй Ильин is marked with CC0 1.0).

Three groups of widely used polymer biomaterials are proteins, polysaccharides, and biocompatible polymer composites (Figure 1). Proteins are macromolecules composed of chains of amino acids. The proteins discussed in this paper are structural proteins such as silk, keratin, and collagen. Silk is by far the most well known protein material, commonly used in many everyday products [27,28]. Keratin is derived from the hair of mammals, including human hair and sheep wool [28,29,30]. Collagen, though the least familiar among the three, is one of the most important components of body tissues. Most raw protein materials must be modified or processed to be suitable for 3D printing [31,32] (Table 1).

Polysaccharides are carbohydrates made up of sugar molecules bonded together. The polysaccharides covered in this paper include chitosan, gellan gum, and cellulose. While chitosan and gellan gum are less commonly known, cellulose is widely used and can be derived from wood or plant cell walls [33,34,35,36,37,38]. Like proteins, polysaccharides also require additives to be 3D printed; however, unlike proteins, polysaccharides are usually not the primary component in the printing material.

The final group of materials are biocompatible polymers and their composites, which are typically used in regenerative medicine, tissue engineering, and drug delivery. These composites are generally biodegradable, biocompatible, and play a crucial role in preventing rejection and minimizing harm when implanted in the body. Unlike the other natural groups, some synthetic biocompatible polymer composites can be printed directly due to their physical properties, such as the ability to melt into viscous liquids at high temperatures or dissolve in solvents. The synthetic biocompatible polymers discussed here are polyhydroxyalkanoate (PHA) and polycaprolactone (PCL) [39,40,41,42].

### 2.1. Silk

Infamous from spiders and sheets, silk is a unique material made up of a strong anti-parallel beta-sheet structure. The beta-sheet endows rigid and strong mechanical properties as the polypeptide chains are tightly stacked to form crystalline regions. The more crystalline the beta-sheet is, the more enhanced the mechanical properties. One important factor necessary to bio print silk is crosslinking. Crosslinking is connecting dispersed water-soluble silk fibroin chains into a continuous water insoluble network [27]. Crosslinking is based on physical or covalent bonds. Under physical bonds the silk fibroin can assemble into beta-sheets through physical crosslinks which consist of tightly stacked segments between or within polypeptide chains via hydrogen bonds. The formation of beta-sheets can be induced by salts, surfactants, heating, and lyophilization. Covalent processes use enzymes including horseradish peroxidase and tyrosinase to crosslink the silk fibroin (SF) [27]. These two enzymes oxidize tyrosine residues to form disulfide or multi tyrosine bonding. Enzyme crosslinking usually produces hydrogels with fine elasticity features. Enzyme crosslinking is limited and hard to control which results in the poor shape of silk fibroin hydrogels. Another way to induce crosslinking is through photo-based methods. Here the crosslinking dynamics are covalently controlled by light intensity. Light is used to excite photo initiators such as riboflavin and lithium acyl phosphonate salt to induce crosslinking. UV light shows a high crosslinking efficiency due to its higher energy; for some applications UV may not be preferred due to its ability to damage cells [5]. A few other methods for crosslinking are photo-oxidation, persulfate, and singlet oxidation. To be printed, silks must be turned into a hydrogel, as hydrogels form 3D crosslinked hydrated fibers which are necessary in the printing process. The addition of methacrylate groups to the amine-containing side groups of a material can be used to make it light-polymerizable into hydrogel [28]. SF can be processed into different forms such as a film, gel, membrane, powder, and porous sponges, and tends to be printed through an inkjet printer.

### 2.2. Keratin

Known to be found in human hair, keratin is a protein found with alpha-helix and (parallel) beta structures that is insoluble in water and most organic solvents. It is mostly correlated with hair care products, but keratin is used for functions not often considered, such as the formation of the tissues for hair, nails, and the outer layer of skin. Keratin gives important structural and protective properties to each of these components. Keratin is very versatile; it is rarely used as the primary components in the objective required such as tissue scaffolding or drug delivery. For 3D printing, keratin has impressive mechanical properties such as high compressive and dynamic properties, swelling capacities, cytotoxicity, and microstructural characteristics [28]. The disulfide bonds of cystine that form during crosslinking allow for greater stability and low solubility [29]. Like silk, keratin needs to be 3D printed in a hydrogel form as it is mainly used for tissue engineering and regenerative medicine applications. Keratin needs to induce crosslinking before being 3D printed and one way to do that is using photo-based methods. To induce the crosslinking through photo methods, keratin must be introduced to a molecule that creates free radicals when in the presence of radiation allowing for new bond formation, known as a photo initiator [30]. One is riboflavin-SPS-hydroquinone (initiator–catalyst–inhibitor) that can produce 3D keratin constructs via UV crosslinking in a lithography-based 3D printer (SLA) [28]. Some other forms of 3D printing with keratin are keratin–PLA–chitosan, keratin–cellulose, keratin–glycerol. Some of these methods may not require photo-based crosslinking methods as some of the solutions may induce it. It is helpful to keep in mind that keratin may not be the main ingredient in these other solutions.

### 2.3. Collagen

Collagen-containing hydrogels are currently the most popular cell scaffold and material for tissue engineering. They have a very high chance of clinical success due to collagen biomaterials being used for a long duration of time, showing viability, immunogenicity, and compatibility. Collagen is a protein that is made of three polypeptide chains. Most collagen hydrogels are produced from type I collagen, which belongs to a group of fibril-forming collagens which have three alpha-helices that form a triple-helical structure [31]. Under specific conditions (neutral pH and 37 °C), collagen molecules automatically form into fibrils, and the collagen solution forms into a hydrogel. The main issue associated with using collagen as a constructive material is its mechanical properties; therefore, most studies show the use of collagen solutions in low concentration (5–10 mg/mL). When using a supportive hydrogel for 3D bioprinting with a collagen bioink, the whole process occurs inside of the secondary hydrogel [31]. Collagen hydrogels are able to be printed through an extrusion method into special support baths. Collagen must also undergo a physical or chemical crosslinking method before printing is allowed; this is conducted through similar ways to silk and keratin [32]. This may be performed through chemical use or photo methods such as UV light. Collagen bioinks must contain additives as printing on its own will not suffice. Collagen bioprints are mainly used for cells, tissues, and organ components. All of these tend to be soft and under gravity can lose their structure quickly. Collagen printing, or tissue/organ printing, may benefit from being printed in a low-gravity environment such as our planet’s orbit, more specifically at the International Space Station.

### 2.4. Chitosan

The 3D printing of chitosan hydrogels has attracted wide interest because of their excellent biocompatibility, antibacterial activities, biodegradability, zero toxicity, and low cost. Chitosan is a linear polysaccharide which consists of several monosaccharides in a straight chain. However, 3D-printed chitosan scaffolds tend to lack the strength required to be a successful construct and therefore require additives. Chitosan is soluble in acid aqueous media at pH 5–6 at 24 °C; sol–gel transition can occur by known gelation techniques such as crosslinker-mediated, polyelectrolyte-complexed, or self-assembled gelation [33]. Another suggested way to increase the strength is to dissolve the chitosan into an alkali aqueous solution. The chitosan is stable at low temperatures (5 °C), but once heated, the chains self-assemble which leads to gelation [33,34]. The direct ink writing method was developed to print high-strength chitosan hydrogels. This writing method comprises a temperature-controlled nozzle that is loaded with the chitosan solution. Once the solution is heated and gelation is assembled, the chitosan is printed directly into heated deionized water to keep the print stable [34]. This method has been seen to produce a chitosan hydrogel scaffold with good quality and high strength. Besides tissue scaffolding, chitosan can also be used for a biosensor interface design.

### 2.5. Gellan Gum

Originally used as a food thickener, gellan gum is being repurposed for 3D printing biomaterials. Gellan gum is classified as a water-soluble linear anionic polysaccharide. It is derived from fermenting sugar with *Pseudomonas elodea*, a bacterium. Gellan gum is an option for many tissue engineering applications due to its easily tunable properties and controllable degradation rates, as well as its mechanical properties such as heat and acid stability. Gellan gum’s use in tissue engineering is primarily aimed at wound dressings, artificial cartilage, and bone osteogenesis. As of now, wound dressing is the most promising due to gellan gum’s ability to crosslink with other chemicals to enhance healing properties as well as its soft texture. One way of increasing crosslinking without using chemicals is through photo methods such as UV crosslinking [35]. Gellan gum’s compatibility to be 3D printed has caught the eyes of researchers as it allows for the control of porosity in the prints. Porosity is a crucial factor to control due to its influence on the rate of degradation and its effect on nutrient diffusion, cell infiltration, and loaded drug delivery rates. The porosity and surface area of gellan gum can be changed to customize to the individual needs of a patient or product. Gellan gum powder, Gelrite, is used to fabricate hydrogels. To be able to print Gelrite you must mix it with ultrapure water and then heat it in a water bath. Following this, Gelrite must be maintained at 53 °C for printing [36]. The hydrogel is then poured into a syringe keeping it at the maintained temperature. This printing process was performed using an incredible bioprinter. This 3D bioprinter was created for tissue engineering and uses pressure to extrude the hydrogel into a scaffold while curing it with UV light [36].

### 2.6. Cellulose

Cellulose is the most abundant, renewable, inexpensive, and readily available polysaccharide in the world, with derivations from plant fibers and wood. Cellulose is tough, fibrous, and water-insoluble. Certain forms of cellulose, such as nanocrystals (CNCs), nanofibers (CNFs), and bacterial nanocellulose (BNC) have attracted great attention for biomedical applications thanks to their high surface area, high mechanical strength, tunable surface chemistry, excellent biocompatibility, cellular recognition, and biodegradability [37]. CNCs have highly crystalline cellulose nanostructures while CNF and BNC are long flexible cellulose fibers. Each form has unique mechanical properties which allow for the integration of each form for different needs. BNC is a great reinforcement agent in hydrogels because of it mechanical strength and tunable surface chemistry properties. CNF hydrogels allow for stability and CNC allows for a high viscosity and storage modulus [37].

Like other polysaccharides and proteins, cellulose needs additives to successfully improve printability and various properties. Alginates, hyaluronic acid, and gelatin have all been used to increase printability. CNF is the type of cellulose that is most compatible with printing, CNC shows poor shear thinning and gelling properties, while BNC is mostly used as an additive [38]. There are challenges with printing just cellulose due to cellulose thermally decomposing before it becomes flowable, which is another reason why it is often used as an additive [38]. This is due to the hydrogen bonds that exist between the cellulose molecules. A new way of printing cellulose is by printing cellulose acetate, which can easily be dissolved in acetone and then extruded from the printer. When printed, the acetone evaporates leaving the cellulose acetate, which solidifies. The print is then treated with a sodium hydroxide mixture [38]. This process is performed at room temperature, eliminating thermal decomposition. Cellulose can be used for biosensors, tissue scaffolding, wound dressing, and artificial skin.

### 2.7. Polycaprolactone

Polycaprolactone (PCL) is a synthetic biocompatible polymer that is known for its easy manipulation, biocompatibility, and stability. This synthetic polymer is popular with bone tissue engineering, but due to its hydrophobic makeup, it decreases cell adhesion and bioactivity when used. Therefore, PCL must contain an additive for biocompatibility. A fine solution to this problem is hydroxyapatite (HA), which increases hydrophilicity, improves cell attachment, and increases mechanical properties [39]. Hydroxyapatite is also a mineral component of bones and forms tough bonds with the original bone. In order to print PCL, it must be dissolved in dichloromethane (DCM) and then an HA powder is introduced. The powder is stirred in while the temperature is held at 35 °C to allow the solvent to evaporate. Immediately after this process, the PCL/HA mixture is entered into the printer. The PCL/HA mix is used in a fused deposition modeling (FDM) 3D printer. This is a standard 3D printer that uses a filament (PLA/HA) that is fed into a heated nozzle which then extrudes the filament for the desired shape with uniformly distributed rectangular pores. The PLA/HA particles are evenly distributed, and the HA particles are agglomerated which allows for greater tensile and flexural strength [40]. Besides bone and tissue engineering, PCL’s numerous mechanical advantages allow trachea substitutes to be created.

### 2.8. Polyhydroxyalkanoate

Polyhydroxyalkanoates (PHAs) are a group of biocompatible polyesters synthesized by microorganisms under balanced growth conditions. One microorganism that PHA is derived from is *Cupriavidus Necator*. PHA has a vast number of impressive properties such as high mechanical properties, cytocompatibility, surface properties associated with cell adhesion, and controlled biodegradability rates. PHA allows customized design due to the quick manufacturing of it. The uses of PHA are mainly focused on drug delivery, vessel stenting, and tissue engineering. One composite of PHA is PHA/PF, where PF stands for palm fibers. This composite shows impressive mechanical properties such as tensile and flexural strength. Fused deposition modeling (FDM) allows for this bio-composite to be printed. Its base concept is to extrude a thermoplastic, in this case the PHA/PF composite, through one or multiple nozzles [42]. The product would be layered, by which the lower layer would cool and harden almost immediately. PHA also has the ability to be printed without any additives thanks to the innovative selective laser sintering (SLS) process. This process allows for the creation of a controlled porous structure, which is crucial for cell growth support [41]. SLS printing is unique compared with other methods of 3D printing, as this process uses a CO_2_ laser beam that writes “slices’’ onto a series of powdered layers. The basic principle of SLS is the causation of sintering through the application of laser and thermal energy. The heat generated sinters the layered particles together where the powder layer is PHA. Recent studies have demonstrated that selective laser sintering and other additive manufacturing techniques can fabricate PHA scaffolds with a tunable pore architecture, enabling optimized mechanical strength and biological function [41]. Specifically, pore sizes ranging from 200 to 500 µm have been shown to support osteogenic differentiation and facilitate neovascularization, improving integration with host tissue [43]. All of these advances highlight the potential of 3D-printed biomaterial scaffolds as promising biomaterials for regenerative medicine and tissue engineering applications.

## 3. Three-Dimensional Printing Theory and Methods

### 3.1. Printing Development

In the 2D printing sphere, inkjet printing is the most prolific. Developed in the 1950s as a replacement for the time-extensive and inaccurate dot printer, inkjet printers were capable of faithfully reproducing images with a higher quality and precision within twenty years [44]. Inkjet printers work through the layering of ink through a nozzle that sprays onto the surface below while printing. Around the same time, the laser printer found its way into development as an alternate solution to the new inkjet printer, with both hitting the commercial market in the mid-1980s [45]. While both were originally made for use in industrial settings, the inkjet printer is the most common household printer type in the United States, with laser printers close behind. The transition from an expensive but necessary industrial machine, through advanced engineering and scaled pricing through mass production, to a technology found throughout millions of homes, schools, and businesses in the United States alone in a relatively short time shows how sound and useful products can rapidly evolve.

It was only logical that after working successfully in the 2D print space the next evolution was to add an additional dimension to the conventional 2D technology. First conceptualized and iterated in the 1980s, 3D printing was a natural choice for taking ideas out of a program and directly creating precise structures for consumers right in front of them, eliminating the need to search for and order new designs as well as making a more efficient solution to conventional object creation. The first 3D printer, invented by Charles Hull, was created in the 1980s and named the SLA-1 [8]. This printer was developed to create prototypes quickly and affordably for engineering designs. While this printer was not an inkjet printer, they quickly took over due to their relatively low cost and the fact that they were already capable of printing at picolitre droplet volume out of the box. Because of this, these printers are excellent for use with biological components. It all comes down to resolution. The smaller the unit that you can print, the more complex, detailed, and smooth a printed structure can be. Three-dimensional printers seem to be following the same path as their two-dimensional predecessors, with rapidly advancing precision and new capabilities of printing seemingly every day. Three-dimensional printers are now being commonly found in schools and even some tech-savvy households.

### 3.2. Printing Methods

**Figure 2:**
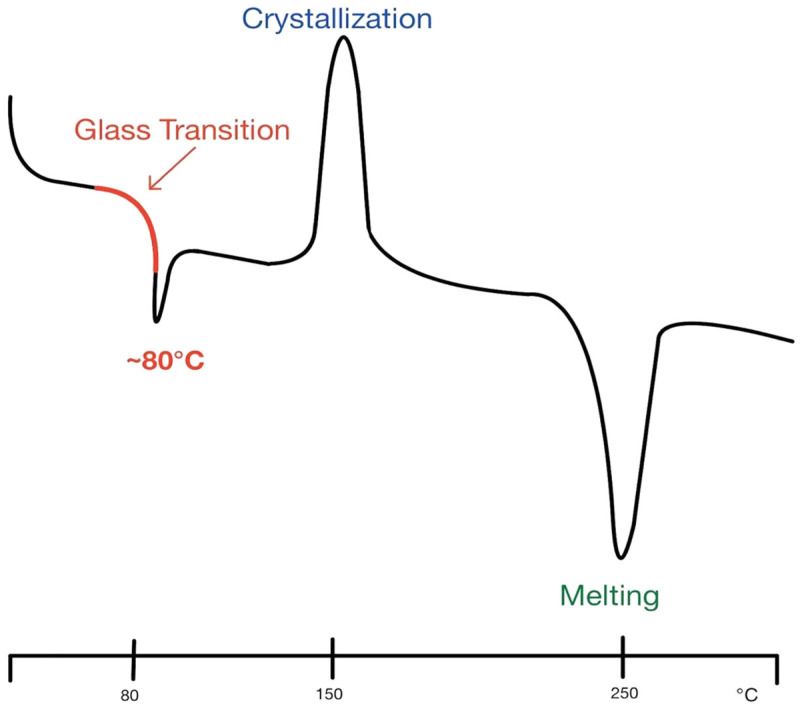
Glass transition of a typical biocompatible polymer. These polymers are conventionally powders printed at glass transition temperature without undergoing crystallization changes for selective laser sintering (SLS).

Printing methods have come a long way from simple additive or stock removal, as was seen in their early phases. While direct filament printing is still performed occasionally in the medical space, most have transitioned to various types of powder printing methods (Figure 1). One of the most common methods is selective laser sintering (SLS). SLS involves heating a powder just above its glass transition level, transforming directly to a solid without passing through the melting phase, allowing for a more precise final product (Figure 2). The downside to this and similar printing methods is the overall waste of the powder [46]. Since the powder not only forms the final product but also acts as a support during the printing process, a lot of printing materials go to waste. There have been some advances in the rapid prototyping (RP) fields using different types of 3D printers, such as SLS, fused deposition modeling (FDM), and stereolithography (SLA), to make powder paper-like scaffolds through layered sheets [47]. Advances in the 3D printing field can originate from a variety of seemingly unrelated fields. RP is a process that has primary uses in the manufacturing of automotive and aerospace vehicles. Future advancements can come from any other field, even one that may not seem associated with that of 3D bioprinting.

### 3.3. Printing: Solution Drying

When printing, materials are typically made into solutions or materials are heated to a liquid phase to be easily extruded through nozzles. Solution drying techniques have typically been air drying, UV crosslinking, or chemical crosslinking, such as using the Ca^2+^ ion drying agent. With regard to chemical drying agents, Ca^2+^ and Cu^2+^ ions have both been used extensively in hydrogel preparations. For 3D-printed drug release usage, freeze drying has been shown to induce faster drug uptake in the final drug delivery product. This is due to the more porous final drug delivery printed material that is achieved through freeze drying [48]. Freeze drying allows for a final product that does not require additional chemical additive processing and, in conjunction with current methods, gives designers the ability to tailor the speed of drug release in the body.

### 3.4. Post Printing: Crosslinking

Crosslinking is the process of modifying combinatory materials via a number of methods to bond two or more otherwise-incompatible or insufficiently bonded substrates for further use, such as a 3D biomaterial. Digital light processing (DLP) is a resin-based form of 3D printing that will be discussed in more detail later. DLP typically uses a photopolymerization additive for the curing process that allows the material to solidify via ultraviolet light [49]. This is a form of crosslinking necessary to print with the primary biomaterial. A good example is the silk fibroin-based bio-ink (Sil-MA) for DLP printing [3]. Inkjet-printable silk-based inks also undergo a similar process to dissolve the inks into an inkjet-printable amalgamation. Finally, and on the leading edge of both 3D printing and in-field use is (in situ) crosslinking. In situ crosslinking refers to a DLP printing technique that uses a Norborne-enhanced hyaluronic acid ink that is curable via light polymerization just before the layer is deposited (printed) onto the patient tissue. This occurs in real time, simultaneously, and stepwise in the printing process and is able to achieve a resolution of 5 μm [46]. While not yet widespread, post print crosslinking technologies are proving that bioprinting in a first-line medical facility that is soon to be the standard of care.

## 4. Three-Dimensional Printing Technologies

### 4.1. In Situ Direct Printing

In situ direct printing is a horizon technology for some aspects of biomaterials and a daily tool for others. The ability to scan a patient and print them a wound tissue or skin graft directly onto the injury is a technology that is doggedly being pursued [44]. Dental offices and cosmetic dental care are the closest to the widespread implementation of this technology. Photo-cured resins have been used in dentistry since 1978.

The primary use of cured resins in dentistry is in oral surgeries. Though there are other uses of cured resin bioprinting in the field of dentistry, oral surgery covers the vast majority [9]. Trauma is a leading cause of restorative oral surgery. There was a time when a mold of your teeth was made from alginate as you sat and waited for it to harden. Now there are many locations performing 3D scans of your teeth in a fraction of the time. Computer aided design (CAD) software has become commonplace in many dental surgeries [50]. It was only natural for a field that worked with 3D molds and 3D problems to embrace 3D technology so fast. Drilling guides are made from 3D printed models of the mandible for reconstruction. Crown copings and dental framework pieces are just a few of the 3D printing techniques used in dental facilities. In cosmetic dentistry such as the ever-popular translucent orthodontic sets of teeth shifting devices this is at its current apex. A 3D scan is conducted and then, using algorithms and a time dependent equation, a set of progressively changing retainers are 3D printed and sent to the customer.

**Figure 3:**
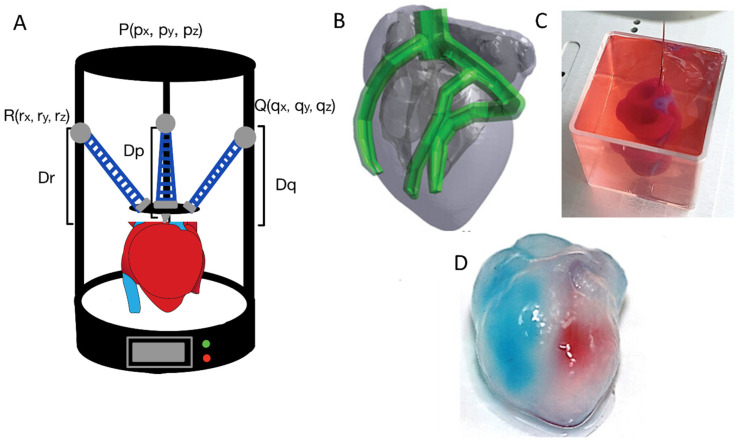
Theory meets reality using 3D printing. (**A**) Theoretical model of a human heart designed for delta printing (adapted heart image sourced from Pixabay.com); (**B**) 3D model of a human heart based on CAD; (**C**) 3D printing a heart within a support bath; (**D**) post print dye infusion to visualize internal chambers and the interventricular septum. (© 2021 Wiley. (**B**–**D**) are distributed under the Creative Commons Attribution License (CC BY), Ref. [51]).

Furthermore, 3D-printed dental implants are used in the dental field, and this technology has been making steady progress. Dental implants have been printed using titanium; however, this is not yet commonplace. Traditional drilled or press-fit implants are usually better suited and more reliable as simple, homogeneous pieces. Complex 3D shapes, however, may be better utilized for irregular bone morphologies, and 3D printing is often useful as an indirect tool in the manufacturing process of pressed or milled interventions. This transition toward patient-specific solutions is further exemplified in the biomedical field, where organ models are being fabricated using advanced 3D printing techniques. One such demonstration involves the direct printing of a human heart model, designed from a CAD file and printed within a support bath to preserve the complex internal features. After fabrication, the heart was removed from the bath, and red and blue dyes were introduced into the left and right ventricles, respectively, to visualize the hollow internal chambers and the interventricular septum, highlighting the model’s anatomical fidelity (Figure 3B–D). This approach underscores the potential of in situ and anatomically accurate printing for surgical planning, educational models, and possibly even the future biofabrication of functional tissue [51].

### 4.2. Inkjet Printers

**Figure 4:**
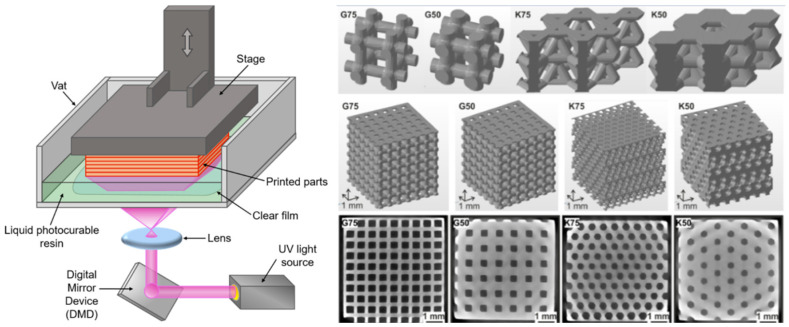
Left: schematic representation of a conventional DLP 3D printer [55] (© 2021 MDPI. Distributed under the terms of the Creative Commons Attribution License (CC BY)). Right: porous scaffolds printed via high-resolution DLP 3D printing for the support of tissue integration and angiogenesis [56] (© 2019 IOP Publishing. Distributed under the terms of the Creative Commons Attribution License (CC BY)).

**Figure 5:**
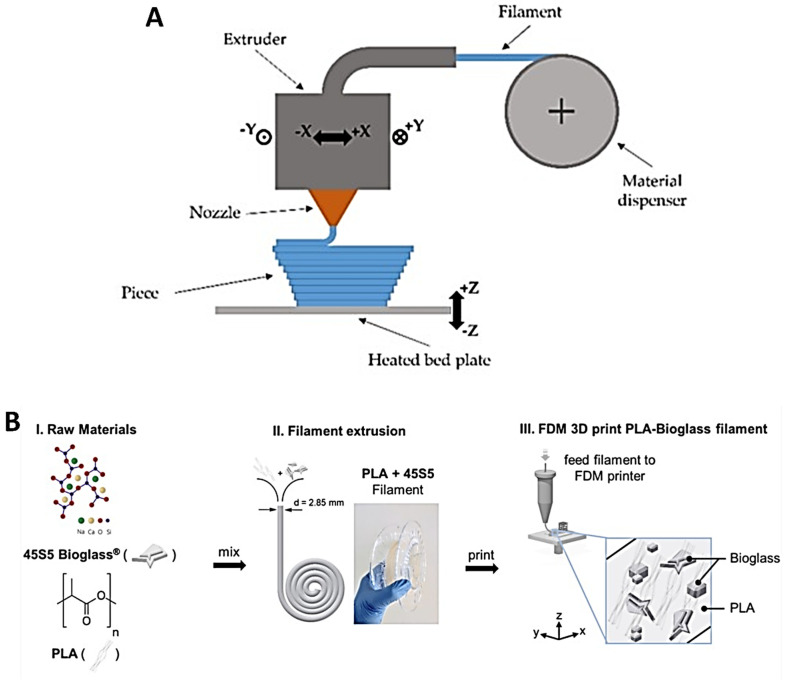
(**A**) Schematic of fused deposition modeling (FDM) 3D printer and its components [57] (© 2019 MDPI. Distributed under the terms and conditions of the Creative Commons Attribution (CC BY) license. (**B**) Step-by-step preparation of a polylactic acid–bioactive glass (PLA-45S5 BG) composite filament [61] (© 2020 Frontiers Publisher. Distributed under the terms of the Creative Commons Attribution License (CC BY)).

**Figure 6:**
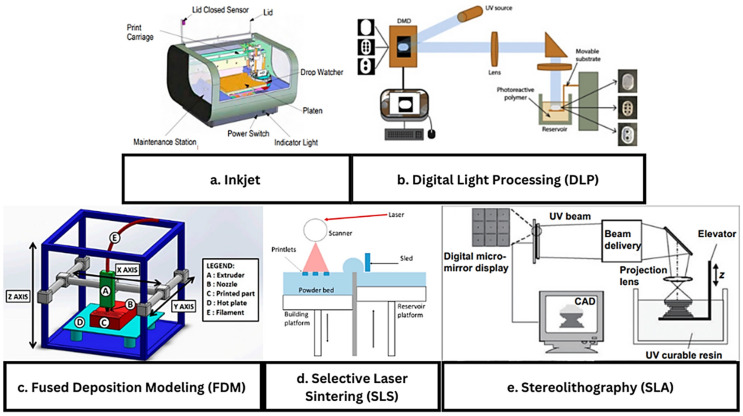
Schematic drawings of 3D printing types typically used in bioprinting: (**a**) inkjet printing (reproduced with permission from ref. [67], Elsevier 2021), (**b**) digital light processing (DLP) (reproduced with permission from ref. [68], Elsevier 2019), (**c**) fused deposition modeling (FDM) (open access distributed under the terms and conditions of the Creative Commons Attribution (CC BY) license by MDPI 2019 [69]), (**d**) selective laser sintering (SLS) (open access distributed under the terms and conditions of the Creative Commons Attribution (CC BY) license by MDPI 2021 [70]), (**e**) stereolithography (reproduced with permission from ref. [71], Elsevier 2005).

Inkjet printing is a layer-by-layer additive printing process. Every printing process uses a slightly different method of printing (Figure 4 and Figure 5). Inkjet is one of the simplest and still one of the most useful printing types thanks to its ability to produce a fine droplet size [52]. In the 3D printing world, like phones and other technological displays, resolution is a key factor. While most commercially available printers are capable of printing below 100 mm, it is now possible to print below the 1 mm range. The widespread use and scaling of this technology will be essential to properly replicate many complex structures in the human body. Inkjet printers build layer by layer by controlling the hot end (inkjet) and maneuvering a base plate (Figure 1e and Figure 6a). In the simplest Cartesian printer, a single nozzle builds layers on a flat plate printing the X and Y cross-sections one Z layer at a time. Multiple heads, temperature-controlled hotplates, and various enclosures that can be employed to control the atmosphere and temperature are also used when needed. Since this technology utilizes multiple printing heads, it is capable of printing over an area rather than just one section. With this, it is also able to print using different materials at once, allowing for material blending [53].

Growing in popularity are slightly more complex printing techniques such as delta printers which have advantages in a few applications, such as creating circular objects and objects with fragile applications. With circular or spherical objects, the hot end can pivot and print on an acute or obtuse angle as needed [54]. This allows for the better building of sloping spherical applications. Fragile applications are best printed with a delta printer if possible because many delta printers do not need a moveable printer bed (hot plate) as all three arms are able to maneuver the print head or heads about the XYZ plane without the need to shift the object. This allows the printed object to remain motionless on the hot plate after printing. Also, it is a common misconception that the delta printer uses a different coordinate system. It still prints XY in Z slices but translates those coordinates to its print head arms (usually three) with trigonometric conversions.

### 4.3. Digital Light Processing

Digital light processing (DLP) was created in 1987, originally for use in cinema, but 30 years later, its application in 3D printing has proven highly successful in advancing the field (Figure 1b and Figure 4). DLP is an additive manufacturing technique in which a projector cures a material (usually a photopolymer) resin layer by layer (Figure 6b). The selected areas are cured and solidified, while the surrounding regions remain untouched. Once one layer has finished curing, the object is raised by the height of one layer, and the process is repeated. This method is particularly effective for fabricating highly intricate and detailed structures that are difficult to achieve with other additive manufacturing techniques. Its ability to produce microscale architectures with high spatial precision, from the micrometer to the sub-micrometer range, makes it especially advantageous for applications requiring a fine structural resolution, surpassing the efficiency and accuracy of methods based on localized, point-by-point curing [55].

New research into digital light processing (DLP) additive manufacturing has shown promising results in improving the fabrication of complex structures for tissue engineering. In one specific example, a silk fibroin-based bio-ink (Sil-MA), produced via methacrylation using glycidyl methacrylate (GMA), has been applied to DLP printing for tissue and organ engineering. In the lab, Kim et al. were able to produce organs and tissues with tubular components such as the trachea and vascular networks, as well as organs like the heart and lungs, all with good mechanical integrity, using this Sil-MA bio-ink in a 3D DLP printer [26]. The benefit of using bio-inks with DLP printing systems is convenience, as one can print very complex structures with multiple branches (e.g., the capillary networks of the heart) in a single procedure. The structures produced in the lab were not only stable but also capable of being layered up to 45–50 mm, significantly higher than the layering capability of alternative hydrogels [52]. In addition to high-resolution fabrication, DLP printing also enables the creation of porous scaffolds, which hold significant potential for applications such as tissue integration and angiogenesis (Figure 4) [56].

### 4.4. Fused Deposition Modeling

Fused deposition modeling (FDM), also known as fused filament fabrication (FFF), is a 3D printing process in which filaments of thermoplastic materials are fed through a heated printer head controlled by a computer (Figure 5) [57]. The filament is heated into a semi-liquid state and then extruded layer by layer, with the layers fusing together to form the final object (Figure 6c). The print head can be equipped with different types of heating nozzles depending on the material being used. These thermoplastic filaments can exhibit a wide range of properties, including transparency, insulation, conductivity, and even magnetic capabilities. This versatility, combined with a relatively low cost, ease of production, and sustainability, has made FDM one of the most popular 3D printing technologies. However, since most materials used in FDM are petroleum-based and not environmentally friendly, research into bio-based alternatives is increasingly important. The most commonly used bio-based materials are polylactic acid (PLA) and polyhydroxyalkanoate (PHA) [58].

In biomedical settings, FDM (Figure 1d) has taken on a prominent role due to its versatility. Recent applications include the fusion of composites to mimic tissues and organs for use in in vivo environments with no toxic effects, the fabrication of microfluidic and reaction ware devices for chemical synthesis, and the creation of porous scaffolds using PLA foam [59]. FDM/FFF has demonstrated its utility in biomedical engineering, particularly as a fast and cost-effective additive manufacturing method compared with more time-consuming and expensive lithographic techniques [60].

### 4.5. Selective Laser Sintering

Selective laser sintering (SLS) 3D printing is known for its ability to produce high-resolution prints at a relatively faster rate than other printing methods, such as fused deposition modeling (FDM). SLS printing typically yields parts with a superior structural integrity and mechanical strength. One of the biggest advantages SLS offers is its ability to print without the need for support structures during fabrication [62]. SLS operates in a bottom-up fashion, dividing the part layer by layer, which allows multiple parts to be printed simultaneously within the same build space. The process begins by spreading a thin layer of polymer powder across the print surface in the desired shape. A laser then selectively sinters the powder particles, binding them together to form a solid structure (Figure 1a and Figure 6d). After each layer is completed, a new coating of powder is applied on top to form the next layer. However, the range of materials that can be used with SLS is more limited compared with other printing methods, as not all polymers can be converted into powders or sintered effectively. The most commonly used material for SLS is polyamide-12, widely known in industry as nylon 12. Another drawback of SLS is the extensive post processing it often requires. While SLS is frequently used for prototyping due to the durability and mechanical strength of the prints, the resulting surface finish often lacks the refinement required for end-use products. If used for final products, additional surface treatment techniques, such as sandblasting, post processing coloring, or surface smoothing, may be necessary to ensure functionality and appearance.

In the field of biopolymer printing, SLS is primarily used for tissue engineering applications, as it can fabricate scaffolds layer by layer. This enables the creation of complex structures with properties conducive to cell infiltration and sufficient mechanical strength to mimic various tissues in the body. SLS has also gained traction in bioprinting through the use of biocompatible polymers such as polycaprolactone (PCL), hyaluronic acid (HA), and even titanium [63]. Due to its high mechanical performance, SLS has recently been applied to bone tissue regeneration, where it helps replicate the properties of native bone and supports the differentiation of osteogenic stem cells into the desired cell types [64]. These printed structures can also be made porous, which is essential for nutrient and oxygen diffusion to support healthy cell growth.

### 4.6. Stereolithography

Stereolithography (SLA) is a form of additive manufacturing 3D printing that is rapidly gaining popularity for its ability to create prints with high levels of detail and desirable surface properties and finish. The polymer material used in SLA is a resin that cures when exposed to specific wavelengths of light, which are provided by a laser (Figure 6e). This curing process rapidly transforms the liquid resin into a hard plastic or flexible solid. One advantage of SLA printing is the wide variety of polymer resins available, allowing users to select materials that best match the required properties of the final product. Because the printer’s laser has a very high resolution, it can print layer by layer with exceptional precision, resulting in a smooth surface finish. This process is also relatively fast compared with other 3D printing methods. However, SLA tends to produce parts with lower structural strength compared with techniques such as selective laser sintering (SLS). Additionally, SLA-printed products require post processing steps such as curing and washing after printing, and the overall cost tends to be higher. SLA is similar to digital light processing (DLP), but while SLA uses a UV laser to cure the resin, DLP uses a visible light projector [65].

Due to its ability to produce small, detailed prints, SLA has found valuable applications in tissue engineering. Some of the most critical characteristics of a scaffold intended for implantation in biological systems include a high porosity and controllable biodegradability. SLA’s high-resolution capabilities allow for the fabrication of porous structures that facilitate cell infiltration and nutrient distribution. The availability of specific resins enables the customization of material properties, such as degradation rate and mechanical strength, to match the surrounding tissue. Hydrogel printing with SLA, using materials such as polyethylene glycol (PEG), has also been employed for cell encapsulation. These materials offer tunable surface and adhesion properties, which have shown improved cell viability and proliferation [66].

All of these conventional 3D printing techniques, such as fused deposition modeling (FDM) and stereolithography (SLA), primarily employ thermoplastics, resins, or composites, and are optimized for structural accuracy, mechanical strength, and rapid prototyping across industrial sectors [72]. In contrast, 3D bioprinting utilizes bio-inks, often hydrogels laden with living cells, biomolecules, or biopolymers, requiring the stringent control of temperature, sterility, and biocompatibility to ensure cell viability and functional tissue formation [25,73]. While conventional 3D printing excels in manufacturing complex, durable, and high-resolution structures, 3D bioprinting focuses on mimicking the native extracellular matrix, enabling applications in regenerative medicine, drug screening, and organ-on-chip models [23]. Despite these significant advancements, current biopolymer-based 3D printing and bioprinting technologies face several limitations. The challenges include limited material options that balance printability with biocompatibility, difficulties in achieving high-resolution structures while maintaining cell viability, and scalability constraints for clinical or industrial applications. Furthermore, the lack of standardized bioink formulations and printing protocols hinders the reproducibility of the final products. Future research should focus on developing novel bioinks with enhanced mechanical and biological properties, improving multi-biomaterial printing techniques, and integrating real-time monitoring to ensure construct fidelity and functionality (Table 2). Addressing these issues will accelerate the translation of these technologies into practical biomedical and industrial applications.

### 4.7. Four-Dimensional Printing

Dynamic or 4D bioprinting is an emerging frontier in tissue engineering that involves fabricating constructs capable of changing their shape, structure, or function over time in response to environmental stimuli such as temperature, pH, moisture, light, or biochemical signals [75,76]. Unlike traditional 3D bioprinting, which produces static architectures, 4D bioprinting introduces a temporal dimension enabling the creation of adaptive, self-morphing, and self-healing biomaterials that better mimic native tissue dynamics [77]. Smart hydrogels, shape-memory polymers, and stimuli-responsive bioinks are central to this technology, allowing printed scaffolds to fold, swell, contract, or release therapeutic agents on demand [78,79]. The applications of 4D bioprinting span vascular grafts that can dynamically adjust to blood flow, implantable devices that adapt to tissue growth, and drug delivery platforms that respond to localized disease microenvironments [78]. Despite being in the early development stages, 4D bioprinting promises to revolutionize regenerative medicine by enabling personalized, programmable tissue constructs with enhanced functionality and integration. This evolving technology is expected to have a significant impact on the future of tissue engineering and regenerative medicine.

## 5. Applications

### 5.1. Extracellular Matrix (ECM) Scaffolds

As patients with severe organ failure or defects approach a critical point in their treatment, they may require a transplant. Tissue engineering and 3D-printed organs can meet this need more quickly, affordably, and with greater patient-specific customization than the traditional method of waiting for a donor at the end of a long list. Significant progress is also being made with decellularized scaffolds for tissue applications. This process creates an artificial extracellular matrix that closely resembles bioavailable scaffolds and can mimic their functions. The scaffolds must be porous to allow for nutrient flow and the incorporation of growth factors, which stimulate the growth of new tissue. The scaffold must also be able to adhere to the tissue it is intended to replicate or regenerate. Different scaffold designs are suited to specific applications (Table 3). For example, a stiffer matrix may be required to construct more rigid components such as cartilage or bone, while a more malleable matrix may be better suited for organ regeneration or the cosmetic aspects of tissue engineering [80]. Integrating ECM components into 3D printing has enabled the fabrication of tissue-specific scaffolds with precise architectures, enhancing regenerative potential compared with synthetic materials [81]. Pati et al. pioneered the use of dECM-based bioinks to print 3D tissue analogs, demonstrating excellent cell viability and differentiation capacity [82]. Kim et al. developed vascularized human skin equivalents using ECM bioinks combined with 3D printing, underscoring ECM’s role in promoting vascularization and tissue maturation [83]. Recent advances have demonstrated the use of tetra-acrylate derivatives of multi-armed polyethylene glycol (TetraPAcs) to crosslink thiolated hyaluronic acid and gelatin, producing mechanically robust and printable hydrogels that support high-density cell encapsulation and viability [84]. These advances illustrate the promise of ECM scaffolds in 3D bioprinting to closely mimic native tissue environments, although challenges remain in optimizing bioink printability and mechanical integrity while preserving bioactivity.

### 5.2. Tissue Regeneration

One of the earlier methods for regrowing tissue was developed in the 1990s and was used to help regenerate and replace torn anterior cruciate ligaments (ACLs). A surgeon performs an arthroscopy to remove tissue from the ACL, which contains chondrocytes, or cartilage-producing cells. These chondrocytes are then cultured and expanded until there are enough to be seeded onto a scaffold. Finally, the scaffold is implanted in the body to regenerate tissue using the chondrocytes.

**Figure 7:**
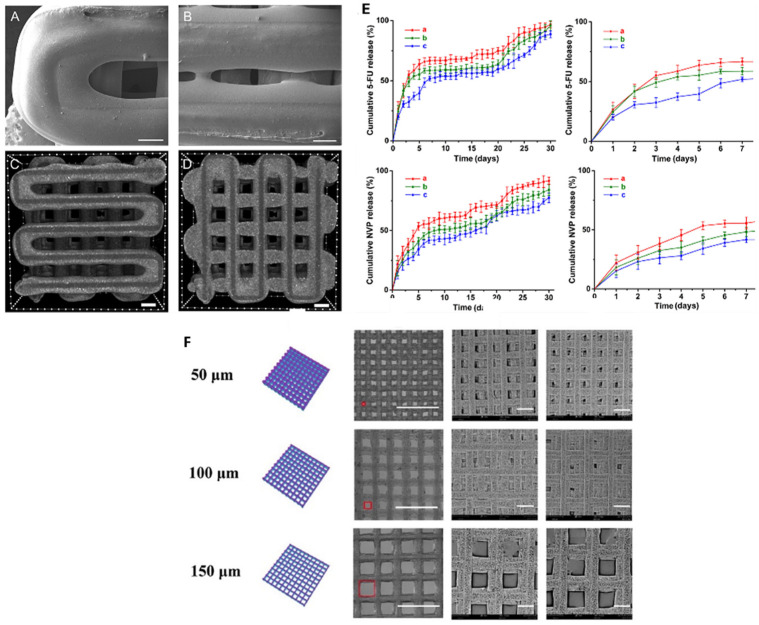
(**A**–**D**) 3D-printed bio-scaffold enabling growth factor attachment and cell proliferation (reproduced with permission from ref. [89], Elsevier 2020). (**E**,**F**) graphs showing how the porosity of the cell scaffold influences the rate of drug release from a drug-eluting stent; pore size of both the stent and scaffold affects the drug delivery rate. Scale bar = 400 μm. (reproduced with permission from ref. [90], Elsevier 2020).

These scaffolds must possess a wide range of properties to successfully recreate living tissue. First and foremost, they must be biocompatible to avoid triggering the body’s immune response. An immune reaction could lead to scaffold rejection and result in inflammation, infection, or other complications. The scaffold must also degrade at an appropriate rate: if it decomposes too quickly, it will not provide sufficient structural support for new cartilage; if it degrades too slowly, the forming cartilage may not develop properly and could become rigid and stiff. Therefore, the correct degradation rate of the scaffold is crucial to its success. Scaffolds can be protein-based, carbohydrate-based, polymer-based, or composed of a combination of these materials. The scaffold’s form depends on its intended function ranging from meshes and hydrogels to more rigid structures designed to support tougher tissue growth. Figure 7 illustrates the pathway through which 3D-printed biomaterials are processed and transformed into biocompatible tissues. Once the printer is ready, with the bed temperature and nozzle size properly adjusted, the printing process can begin. This process involves printing a scaffold that enables cells and growth factors to attach and multiply. To achieve this, the printed material must match the structural requirements of the target cells and provide suitable porosity for cell growth, along with favorable surface charges to enhance the attachment of cells and growth factors. Afterward, the cells and growth factors are deposited onto the material, which is then placed in a bioreactor to promote the development of new tissue.

**Figure 8:**
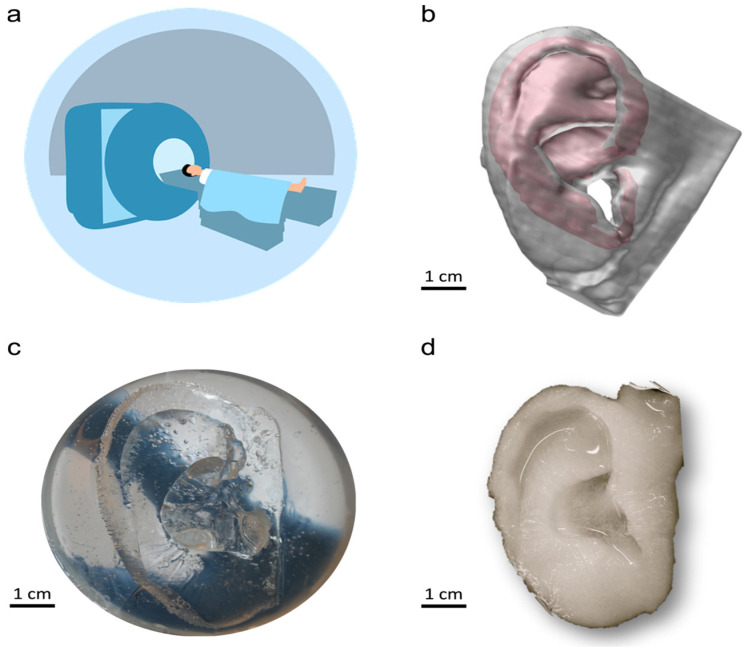
(**a**) MRI of a human ear; (**b**) 3D computer-generated model of the ear; (**c**) replication of the model into a mold; (**d**) culturing of bacterial nanocellulose in the mold to create a usable ear. ((**a**) is open source provided by pixabay.com; (**b**–**d**): reproduced with permission from Ref. [86], Elsevier 2013).

Natural and synthetic biopolymers have been widely used in tissue engineering and regenerative medicine. Biomaterials such as collagen and chitosan, which occur naturally, offer excellent biocompatibility, biodegradability, and cost-effectiveness. Both materials respond well to growth factors and are found in the extracellular matrix. Three-dimensional printing, particularly extrusion-based and inkjet-based methods, is commonly employed in bioprinting for this purpose. Acellular scaffolds containing biological components can be 3D-printed, and bioprinters can also generate cell-laden scaffolds for tissue mimicry and organ repair [63]. Given the national shortage of donor organs and the growing waiting list, the ability to use 3D printing for both organ repair and fabrication could significantly help alleviate this crisis [85]. There are numerous applications for tissue regeneration, ranging from functional organ development to aesthetic reconstruction for trauma victims. An example of the latter is demonstrated in upcoming research on ear cartilage regeneration. Human cartilage tissue was successfully replicated using bacterial nanocellulose (BNC), which was then manufactured into a usable human ear. This was achieved by first acquiring an MRI of a human ear to capture its shape, which was then used to create an accurate mold (Figure 8a,b). From the mold, the BNC was able to be cured to create an ear (Figure 8c,d). The resulting cartilage can be used in both ear reconstruction and meniscus repair, showing strong structural integrity at room temperature [86]. While this artificial skin was developed for aesthetic purposes, it holds great potential for future applications in bioavailable skin. Functional living skin was successfully 3D printed using a bioink known as GelMA/HA-NB/LAP, and digital light processing (DLP) 3D printing was used to produce bioavailable tissue. This bioink is composed of gelatin methacrylate (GelMA), N-(2-aminoethyl)-4-(4-(hydroxymethyl)-2-methoxy-5-nitrosophenoxy) butanamide (NB)-linked hyaluronic acid (HA-NB), and the photo initiator lithium phenyl-2,4,6-trimethylbenzoylphosphinate (LAP). The bioink exhibited biocompatibility, tunable mechanical properties, and strong tissue adhesion. This functional living skin promotes regeneration by mimicking the structure of natural skin. Future work may explore the mass production of functional organs with similar biocompatibility and adhesive properties [87].

Furthermore, 3D bioprinting also has important applications in the manufacturing of bone scaffolds. These printed bone scaffolds included structures that supported both bone formation and microvascular-mimicking channels. This design enhanced bone regeneration and vascular cell growth. By enabling the formation and maintenance of osteoblasts and osteoclasts, the printed bone becomes functionally usable by the body. These scaffolding structures were fabricated using nano-hydroxyapatite (nHA) for vascularized bone growth. Both bone-like physical properties and vascular-like flow profiles were confirmed [88].

### 5.3. Drug Delivery

One of the major applications of 3D printing in medicine is the fabrication of drug-eluting implants and stents. These stents must be biocompatible and capable of gradually releasing drugs over time to ensure continuous and safe therapeutic delivery. Larger porosity within the scaffold can be advantageous in applications requiring high drug dosages over shorter periods, as it facilitates increased drug distribution. The interaction between the drug and the material is also critical, as it influences how and when the drug is released. For instance, a 3D-printed mesh may not release a particular drug as effectively as electrospun nanofibers would with the same compound.

The geometry of the scaffold also plays a significant role in drug release behavior. In some cases, specific geometric constraints are unavoidable, requiring the careful consideration of other variables such as the type of drug used and the scaffold’s porosity. For example, in the case of a stent designed to treat salivary gland disease, the stent must allow for both salivation and proper airflow. Overall, the structure, geometry, drug type, and biomaterial all significantly affect the efficacy of drug-eluting stents.

However, ethical concerns may arise, especially regarding the off-label use of 3D-printed stents. While their effectiveness for approved indications can be validated, physicians sometimes apply these stents in ways not yet approved by the FDA, based on clinical judgment. This creates potential ethical dilemmas and highlights the need for collaboration and clear communication between the engineers who design the devices and the physicians who implement them. Although 3D printing has the potential to greatly increase the availability of such stents, it is essential to address and prevent possible misuse [91,92].

The use of 3D biomaterial printing has practical uses both in vivo and in vitro. Full and complex structures of organs and tissues can be created and tested for various drug delivery applications. Drugs can be tested on 3D body-on-a-chip models to evaluate their effects and side effects before being used on living individuals [93]. The complexities of drug delivery can have life-altering consequences when used in situ, so it is imperative to understand how drugs interact with the body before making them commercially available.

**Figure 9:**
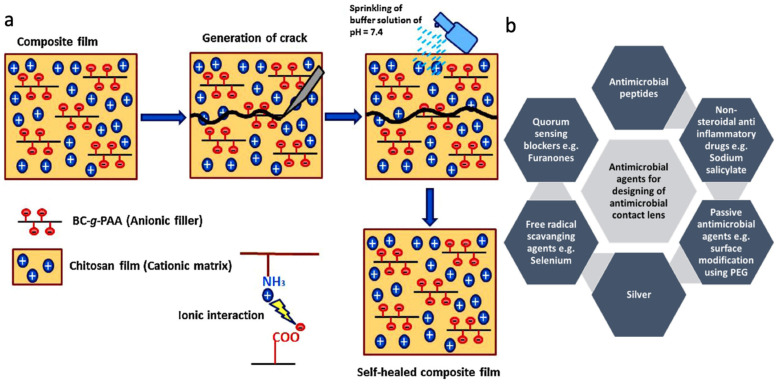
(**a**) Example of a hydrogel that can be used for wound dressing/healing (© 2021 MDPI. Distributed under the terms of the Creative Commons Attribution License (CC BY), Ref. [96]). (**b**) Examples of the various biomaterials that contacts use as antimicrobial. (© 2021 Wiley. Distributed under the Creative Commons Attribution License (CC BY), Ref. [95]).

Drug delivery from hydrogels can be triggered by several mechanisms (Figure 9). Nanocomposite hydrogels can release drugs passively, gradually over long periods, or in response to external stimuli. For example, changes in pH or temperature can cause the hydrogels to shrink or swell. Site-specific hydrogels operate similarly to stimuli-responsive hydrogels and can also react to specific solution compositions. Additionally, hydrogels can act as detoxifiers by absorbing toxins in targeted areas of the body [94].

Drug-eluting contact lenses use drug delivery systems to prevent microorganisms from binding to the lenses and infecting the eye. This is achieved by releasing agents that are toxic to the microorganisms, such as silver or nonsteroidal anti-inflammatory drugs (NSAIDs). These drugs can be released either passively, through slow continuous diffusion, or actively, by disrupting the microbial cell membrane. The most commonly used types are featured in Figure 9b [95]. Drug delivery also has multiple applications in wound healing. One example involves a chitosan film paired with poly(acrylic acid)-grafted bacterial cellulose. Two buffer solutions, at pH 7.4 and pH 5.5, were applied to the film. The ammonia (NH_3_) groups bonded ionically to the carboxylate ions (COO^−^) when exposed to the buffer solutions. This ionic bonding promotes a self-healing mechanism, as shown in Figure 9a [96]. Microneedle drug patches have also been developed using 3D printing. These patches, made with Soluplus (a PVC-PVA-PEG blend) and stainless steel needles, have been used for a variety of treatments such as motion sickness, hypertension, and general pain management [97].

### 5.4. Biosensor

In general, 3D-printed biosensors consist of four major components: the receptor, the interface, the transducer, and the processing equipment. The receptor binds to the desired analyte. The analyte can be described as the target chemical that the biosensor is designed to detect, augment, or suppress. The interface provides an environment for the receptor to bind to the analyte, while the transducer converts the chemical information from the receptor into an electrical signal.

Biosensors allow for the integration of 3D-printed scaffolds and live cells. They are commonly used for medical diagnostics, as they can capture cell cultures and allow them to proliferate in a controlled environment to study disease pathology. Biosensors can also be 3D printed to evaluate various mechanical stresses exerted by extracellular matrices, such as tensile or compressive forces. This enables the in-depth investigation of extracellular matrix properties, as their stiffness significantly affects cell proliferation. It also highlights the importance of porosity in the extracellular matrix (ECM); as the ECM becomes stiffer, it supports more rapid and stable cell migration.

**Figure 10:**
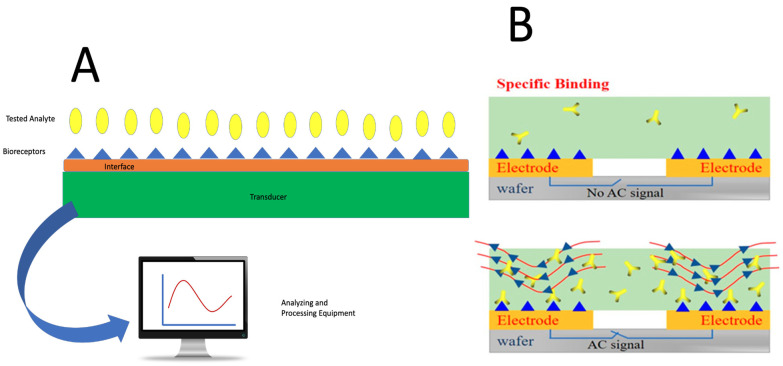
(**A**) The composition of a biosensor (computer monitor image sourced from Pixabay.com, open source). (**B**) Performance of NHS and EDC biosensors, with or without an electric current, in detecting BPA (© 2020 MDPI. Distributed under the Creative Commons Attribution License (CC BY), Ref. [101]).

The processing equipment takes the electrical signal from the transducer and converts it into data that can be interpreted. This process allows for both quantitative and qualitative data to be obtained from biological processes and provides a more detailed understanding of what is occurring within the body’s systems. A system diagram is shown in Figure 10A [98].

Nanoparticles and biocompatible polymers are often combined for use in biosensors. For example, single-walled carbon nanotubes combined with agarose (3,6-anhydro-L-galactopyranose) are fabricated using extrusion printing. This combination is used to selectively pattern the electrical conductivity of deposited structures [99]. NiCo_2_O_4_@PEDOT/RGO biosensors were used to detect H_2_O_2_, a compound that is important to monitor due to its association with diabetes, certain cancers, and Parkinson’s disease. These biosensors demonstrated approximately 95.4% detection effectiveness [100]. Bisphenol A (BPA), which has been linked to heart disease and diabetes in young children, can be detected using N-hydroxysuccinimide (NHS) and 1-(3Dimethylaminopropyl)-3-ethylcarbodiimide hydrochloride (EDC) as crosslinking agents. An alternating current (AC) was applied across the electrodes and the solution to expedite the bonding between BPA and the electrode.

The significance of detecting diseases like Parkinson’s cannot be overstated. With the capabilities of 3D bioprinting, disease diagnostics can be accelerated and potentially used to treat or even reverse degenerative conditions, thereby improving the quality of life for individuals with chronic or progressive diseases. This process is illustrated in Figure 10B [101]. Additionally, biosensors can be used to detect *E. coli* and other pathogens in food. *E. coli* can be identified by monitoring the pH increase caused by ammonia, which results from the urease–*E. coli* antibody conjugate, and a potentiometric alternating biosystem was used to measure pH [102].

The 3D printing of biomaterials also spans a broad spectrum of validation stages. Silk fibroin–nanohydroxyapatite scaffolds have demonstrated in vivo bone regeneration in rat calvarial defect models, highlighting their osteoconductive potential [103]. Gelatin–hydroxyapatite hybrid scaffolds fabricated via microextrusion bioprinting have enabled in vivo articular cartilage repair in large animal models [104]. Gelatin/oxidized-alginate cryogel scaffolds enhanced with hydroxyapatite have demonstrated improved mechanical stability and bioactivity in in vitro studies [105].

**Table 3:** Categories of 3D-printed polymer applications and their uses by physicians and engineers.

3D Printing Application	Desired Function	Key Factors	Optimal Materials	Refs.
ECM Scaffolds	- Create a scaffold to grow new tissue or biomaterial - Must degrade after its function has been fulfilled	- Porous and biocompatible with similar mechanical properties to the body’s ECM - Space for cell proliferation and growth factors	- Alginate - Chitosan - Collagen - Fibronectin - Hyaluronic Acid - Composites - Metals - Ceramics	[106]
Tissue Regeneration	- Create new bioengineered and printed tissue from polymers to create biocompatible working tissue	- Tissue must be able to attach to the natural ECM - Must be biocompatible and able to handle blood flow	- Nanomaterials - Mesenchymal Stem Cells	[107]
Drug Delivery	- Print and develop stents and other inorganic materials that can elude drugs to treat certain diseases	- Implant must be non-toxic to the body - Must elute the drug at the appropriate order (first, second, zero)	- Metals - Drug-Coated Polymers	[108]
Biosensors	- Develop sensors to act as markers for various diseases and bioavailability errors	- Must be able to recognize signals and make affected decisions based on those signals	- Electrically Conductive	[109]

## 6. Limitations and Future Directions

The technique of 3D printing, or additive manufacturing, has revolutionized manufacturing by enabling the creation of complex, customized parts with reduced waste and rapid turnaround times across sectors such as aerospace, healthcare, and consumer goods [110]. Advances in technologies like fused deposition modeling (FDM), stereolithography (SLA), and selective laser sintering (SLS) have expanded the range of printable materials, including polymers, metals, ceramics, and biocompatible bioinks. Bioprinting, a rapidly growing subset, aims to fabricate living tissues by integrating cells with biomaterials, offering promising applications in regenerative medicine [25,72,111].

Although 3D printing enables the rapid fabrication of highly customized and complex structures at relatively low costs, its application in the medical field remains limited by the narrow range of suitable biomaterials. Despite the availability of various materials such as metals, ceramics, polymers, and composites, challenges related to printability, mechanical strength, biodegradability, and biocompatibility restrict their effective use in medical-based 3D printing. In extrusion-based bioprinting, high polymer concentrations are often required to maintain the structural integrity of printed bioinks, resulting in dense hydrogels that hinder cell connectivity and functional integration within the scaffold. For biological scaffolds of moderate size, vascularization is critical for sustaining tissue viability, yet current 3D printing techniques cannot replicate the complex vascular networks necessary for larger, functional organs. While small-scale scaffolds can survive through diffusion, fabricating life-size organs demands advanced strategies to create perfusable channels. To tackle this, researchers have employed sacrificial materials that occupy void spaces during printing, providing temporary mechanical support that is subsequently removed through post processing. Furthermore, limitations arise from the conversion of intricate CADs into machine-readable instructions, which can lead to material discontinuities, as well as from discrepancies between the porosity of the original digital model and the final printed product, impacting the scaffold’s mechanical and biological performance [97,112].

Looking forward, future directions in 3D medical printing focus on developing novel biomaterials that balance printability, mechanical robustness, and biocompatibility, including stimuli-responsive and bioactive materials to enhance tissue regeneration. Advances in multi-material and multi-scale printing aim to integrate vascular networks and cellular components more effectively, moving closer to fully functional organ fabrication. Incorporating artificial intelligence and machine learning into design and printing workflows promises improved precision, defect prediction, and process optimization. Additionally, sustainable and biodegradable materials are being prioritized to reduce the environmental impact. New methodologies that utilize the literature database screening of hundreds of 3D printing cases—following the meta-analyses (PRISMA) guidelines—are also promising, especially when combined with rapidly advancing AI technologies, for identifying optimal solutions tailored to specific 3D printing application needs [113]. The convergence of these innovations will be crucial for translating 3D bioprinting from the laboratory to clinical applications, enabling personalized medicine and regenerative therapies.

## 7. Conclusions

Recent advances in the field of 3D printing have been significant. Biocompatible polymers have made major strides, driven by progress in crosslinking methods, the development of new biomaterials, and improvements in printing techniques. Silk, one of the oldest known biopolymers, has recently been successfully printed using at least two new methods: via methyl acrylate and through biopolymer composite photo initiators. Importantly, not all techniques are new or prohibitively expensive. Excellent work has also been achieved with cellulose, the world’s most abundant and inexpensive polysaccharide, enabling it to be printed through cellulose acetate extrusion methods. Biocompatible polymers such as PCL and PHA are also being successfully used in biomedical applications, particularly in degradable scaffolds and implants.

In the printing domain, PHA has been printed without additives using selective laser sintering (SLS). SLS technology offers a higher precision by printing at the polymer’s glass transition temperature, thereby avoiding the melting phase, which often results in unpredictable outcomes. Silk methacrylate (Sil-MA) has become digital light processing (DLP)-printable thanks to advances in crosslinking research. Both stereolithography (SLA) and fused deposition modeling (FDM) printers offer unique advantages suited to different needs, such as high-resolution detail or superior material strength and structural performance.

Ultimately, the application of these biopolymers and printing techniques is enabling both current and emerging innovations. Three-dimensional printing allows for the rapid production of scaffolds, tissues, drug delivery vehicles, and biosensors. While fully 3D-printed human hearts remain years away, tissue regeneration is at the forefront of technological progress. Component replacements, such as heart valves and tracheal segments, are already being explored to help reduce organ transplant waitlists, while continued research aims to make whole organ printing feasible. As more tissues become printable, more patients and potential organ recipients can benefit. In line with preventive and restorative medicine, 3D-printed biosensors are also driving advances in the early detection of pathogens in food, various cancers, and even neurodegenerative diseases like Parkinson’s. These remarkable developments position 3D printing as a transformative tool in the future of healthcare.

The 3D printing of biopolymers such as silk, cellulose, keratin, chitosan PCL, and PHA have significantly expanded the possibilities for fabricating complex, biocompatible scaffolds and implants. These developments, driven by innovations in crosslinking chemistry, material formulation, and printing technologies, have enabled tailored mechanical properties and biological functionality suited to a variety of biomedical applications. However, several critical challenges remain, including optimizing print resolution and speed, enhancing bioink stability, and ensuring long-term biocompatibility and integration in vivo. Future research must focus on developing multi-material and multi-scale printing strategies, improving vascularization within printed tissues, and advancing regulatory frameworks to facilitate clinical translation. Addressing these challenges will be essential to realize the full potential of 3D bioprinting, ultimately enabling personalized regenerative therapies, complex organ fabrication, and advanced biosensing platforms that transform healthcare delivery.

## Figures and Tables

**Table 1:** Summary of biocompatible materials for medical 3D printing reported in this study, classified by material class, representative examples, and their use as biopolymer-based composites, along with key applications.

Material Class	Materials	Biopolymer Composite	Printability	Mechanical Properties	Cytocompatibility	Applications
Proteins	Silk fibroin (SF)	e.g., SF with methacrylate groups, SF hydrogels	Photo-crosslinkable; UV-curable; good print resolution [27,28]	High tensile strength, elasticity [27]	Excellent biocompatibility, promotes cell adhesion [28]	Porous materials, tissue scaffolds printed via UV crosslinking
	Keratin	e.g., Keratin–PLA–Chitosan, Keratin–Cellulose, Keratin–glycerol	Printable via SLA-based UV crosslinking; blendable with PLA, chitosan [29,30]	Moderate mechanical strength; flexible	Good cytocompatibility; supports regeneration [29]	Tissue scaffolds, drug delivery, regenerative medicine; SLA-based UV crosslinking
	Collagen	Blended with supportive hydrogels for 3D printing	Direct ink writing; requires additives for printability [31,32]	Moderate mechanical strength, biodegradable [31]	Excellent biocompatibility, antimicrobial [32]	Cell scaffolds, tissue/organ components; printed in support baths
Polysaccharides	Chitosan	Requires additives; printed via direct ink writing into heated water	Printable in support baths; blends with hydrogels [33,34]	Low mechanical strength alone; often combined for reinforcement [33]	Excellent cytocompatibility, native ECM component [34]	Tissue scaffolding, biosensor interfaces
	Gellan gum	Crosslinked with chemicals or UV	UV crosslinkable; printable hydrogels [35,36]	Soft mechanical properties; tunable via crosslinking [35]	Good cytocompatibility [36]	Wound dressings, artificial cartilage, bone osteogenesis
	Cellulose (CNF, CNC, BNC)	As additive or modified cellulose acetate	Printable as additive or modified derivatives [37,38]	Variable mechanical properties; generally high strength [37]	Biocompatible; supports tissue growth [38]	Biosensors, tissue scaffolding, wound dressing, artificial skin
Synthetic Biocompatible Polymers	Polycaprolactone (PCL)	e.g., PCL + hydroxyapatite	Good printability with the composites [39,40]	Improved mechanical properties [39,40]	Broad applications in bone tissue engineering [40]	Bone scaffolds, trachea substitutes
	Polyhydroxyalkanoates (PHA)	e.g., PHA + palm fibers	Porous scaffolds printed by SLS [41]	Good mechanical properties [42]	Biocompatible [42]	Drug delivery, vessel stents, tissue engineering; FDM and SLS printing

**Table 2:** Three-dimensional bioprinting types organized by advantages and disadvantages with associated printable biomaterials discussed in review.

Printing Type	Printing Method	Advantages	Disadvantages	Biomaterials	Refs.
Inkjet	Layer-by-layer deposition	- Multiple print heads - Smooth finish - Spread area printing - No post processing	- Low material properties - Limited materials	- Silk (composite) - Cellulose - Gellan gum	[44,52,67]
Digital Light Processing	Photopolymerization (resin curing)	- Many materials - Quick printing - High accuracy and quality	- Post curing required - Lower structural properties - Necessary post processing	- Silk (composite) - Gellan gum	[26,46,52,68]
Fused Deposition Modeling	Extrusion	- Low cost - Material variety - Basic printing style	- Lower mechanical properties - Necessary post processing - Frequent machine issues - Long print time	- PCL - PHA	[58,59,60,69]
Selective Laser Sintering	Powder sintering	- No supports - High material properties - Relatively lower cost - Fast print time	- Necessary post processing - Low material availability - Brittle	- PCL - PHA - Cellulose	[41,62,63,64,70]
Stereolithography	Photopolymerization (resin curing)	- Smooth finish - Material variety - Quick printing - High accuracy	- Higher cost - Post curing required - Lower structural properties	- PCL - PHA - Cellulose - Gellan gum	[65,66,74]
